# Ultra-high open-circuit voltage of tin perovskite solar cells via an electron transporting layer design

**DOI:** 10.1038/s41467-020-15078-2

**Published:** 2020-03-06

**Authors:** Xianyuan Jiang, Fei Wang, Qi Wei, Hansheng Li, Yuequn Shang, Wenjia Zhou, Cheng Wang, Peihong Cheng, Qi Chen, Liwei Chen, Zhijun Ning

**Affiliations:** 1 School of Physical Science and Technology, ShanghaiTech University, Shanghai, 201210 China; 2 i-Lab, CAS Center for Excellence in Nanoscience, Suzhou Institute of Nano-Tech and Nano-Bionics, Chinese Academy of Sciences, Suzhou, 215123 China

**Keywords:** Climate change, Environmental impact

## Abstract

Tin perovskite is rising as a promising candidate to address the toxicity and theoretical efficiency limitation of lead perovskite. However, the voltage and efficiency of tin perovskite solar cells are much lower than lead counterparts. Herein, indene-C_60_ bisadduct with higher energy level is utilized as an electron transporting material for tin perovskite solar cells. It suppresses carrier concentration increase caused by remote doping, which significantly reduces interface carriers recombination. Moreover, indene-C_60_ bisadduct increases the maximum attainable photovoltage of the device. As a result, the use of indene-C_60_ bisadduct brings unprecedentedly high voltage of 0.94 V, which is over 50% higher than that of 0.6 V for device based on [6,6]-phenyl-C61-butyric acid methyl ester. The device shows a record power conversion efficiency of 12.4% reproduced in an accredited independent photovoltaic testing lab.

## Abstract (web summary)

Despite the lower device efficiency, tin perovskite based solar cells are preferred choices compared to lead-based counterparts due to much lower toxicity. Here Jiang et al. use a fullerene derivative to greatly suppress carrier interface recombination and obtain record high cell efficiency of 12%.

## Introduction

Halide perovskites are rising as star materials for next-generation solar cells. However, the state of the art lead perovskite still needs to be upgraded since: firstly, the heavy metal character of lead may cause an environmental concern^1,2^; secondly, according to Shockley–Queisser limitation, the highest efficiency of lead perovskite is <31% due to its large bandgap (1.55 eV)^3^. The development of alternative perovskite materials is therefore highly desirable. Tin perovskite is regarded as an ideal candidate due to its narrow bandgap and environmental benign character^4^. Up to now, tin perovskite solar cell (PSC) demonstrates the highest efficiency among all kinds of lead-free perovskites^5^.

Despite the rapid development of tin PSCs, the efficiency is much lower than lead perovskite nowadays. One critical factor is the extremely low open-circuit voltage. High defect density in the film is one important factor contributing to the low voltage^6^. Some methods are implemented to reduce defect density of tin perovskite, e.g., addition of reductive additives^7–9^, and perovskite dimensionality manipulation^10–12^, which increased the voltage of the device from 0.3 V to 0.6 V (ref. ^13^). However, compared to its bandgap (1.35 eV), the voltage loss of tin PSCs is still over 0.7 V, much larger than that of lead perovskite. It is highly desirable to clarify the mechanism for the low voltage of tin PSCs and find solutions.

Another possible factor for the low voltage of tin PSCs could be the deep Lowest Unoccupied Molecular Orbital (LUMO) energy level of electron transporting layers (ETLs), e.g., [6,6]-phenyl-C_61_-butyric acid methyl ester (PCBM)^14,15^ and buckminsterfullerene (C_60_)^16–18^, which limited the maximum attainable photovoltage^19^.

Here, we introduce indene-C_60_ bisadduct (ICBA) as ETL for tin PSCs to replace the generally used PCBM. ICBA with shallower energy level brings a larger maximum attainable voltage and it suppresses iodide remote doping that reduces interface carriers recombination. As a result, the use of ICBA as ETL improves the voltage of the device to 0.94 V, which is significantly higher than that of the device using PCBM as ETL (0.6 V). The device based on ICBA demonstrates an unprecedentedly high efficiency of 12.4%, which is almost 30% higher than the highest efficiency reported up to now^20–22^. Moreover, the device shows excellent shelf stability by maintaining 90% of the initial efficiency for over 3800 hours. This work clarifies the mechanism for the low voltage of tin PSCs, and presents a strategy to increase the voltage and efficiency of tin PSCs.

## Results

### Perovskite film structure

Perovskite films are prepared using a typical anti-solvent method via a one-step process (details can be found in Methods section). We tune the concentration of PEA (PEA = C_6_H_5_CH_2_CH_2_NH_3_^+^) in precursors to get the PEA_*x*_FA_1−*x*_SnI_3_ (FA = HC(NH_2_)_2_^+^) perovskites (abbreviated as PEA*x*, *x* = 0 and 15). Films are prepared on poly(3, 4-ethylenedioxythiophene):poly(styrene sulfonate) (PEDOT)^18^ substrates to control the nucleation and orientation of perovskite. NH_4_SCN (SCN) is used as additive to modify perovskite film growth.

**Fig. 1:**
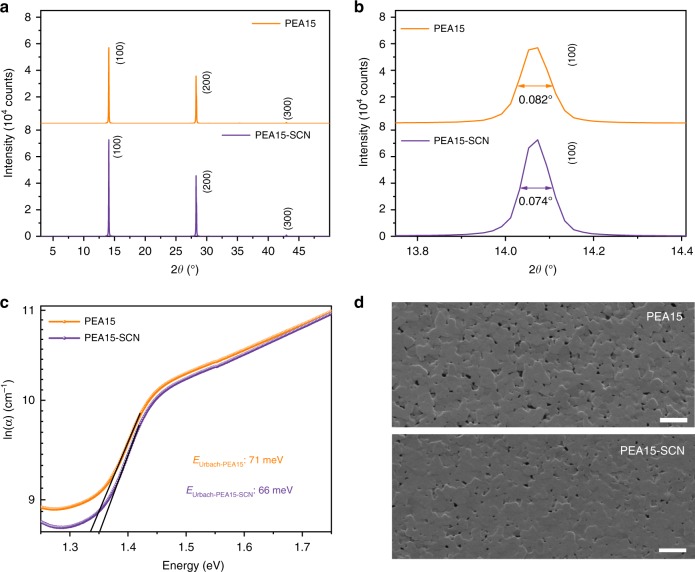
Perovskite film characterization. **a** XRD spectra of perovskite films and **b** the corresponding FWHM values of (100) peak. **c** The Urbach energy of perovskite films. **d** SEM images of perovskite films. The scale bar is 1 µm.

We characterized the crystal structure by X-ray diffraction (XRD). The diffraction peaks of the perovskite film with NH_4_SCN (PEA15-SCN) show a higher intensity (Fig. 1a) and a smaller full-width half-maximum (FWHM) value (Fig. 1b), indicating a larger domain size for PEA15-SCN film.

We then studied the microstructure of perovskite film by grazing-incidence wide-angle X-ray scattering (GIWAXS). The strong diffraction spot in 90 degree shows that PEA15-SCN film is perpendicularly grown on the substrate along [100] direction^23,24^ (Supplementary Figs. 2–4). Both films with and without SCN grown on PEDOT show similar diffraction spots from single layer and double layer structures at both small and large grazing-incidence angles (Supplementary Fig. 3). This is completely different from the image of hierarchy structure grown on NiO_X_ substrate^12^. We conclude that low dimensional structures homogeneously distribute in the film, which can be ascribed to the higher binding energy of PEA and FA molecules on PEDOT substrate, as calculated by density functional theory (DFT) simulation (Supplementary Fig. 6, Supplementary Table 1).

We performed spectroscopy characterization to study the structure of the films further. The absorption edge of the film based on PEA15-SCN (905 nm) is quite close to that of three-dimensional (3D) structure PEA0 (917 nm; Supplementary Fig. 7a), implying the presence of 3D-like structures in the film. In combination with GIWAXS measurement, it can be speculated that part of PEA molecules are consumed for constructing two-dimensional (2D) and 2D-like structures, which are mixed with 3D structures in the film (Supplementary Fig. 8). The absorption spectrum of PEA15-SCN film demonstrates a lower Urbach energy of ~65 meV (Fig. 1c), which indicates the reduced defect concentration with the addition of NH_4_SCN (refs. ^25,26^). Scanning electron microscope (SEM) images of PEA15 and PEA15-SCN films are similar and both of them show smooth morphology (Fig. 1d).

### Device structure design

**Fig. 2:**
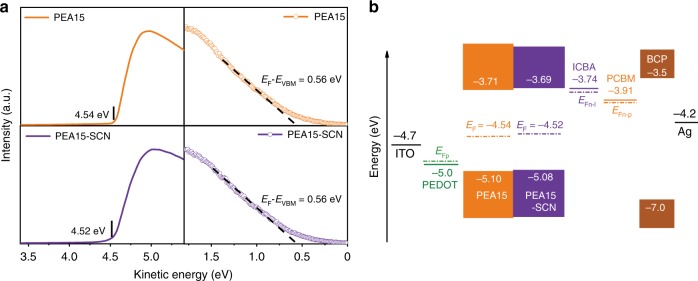
Band structure of tin PSCs. **a** UPS spectra of secondary electron cutoff and valence band of perovskite films. **b** Schematic illustration of energy levels. Dashed lines represent the quasi-Fermi level of ICBA (*E*_Fn–I_), PCBM (*E*_Fn–P_), and PEDOT (*E*_Fp_).

We measured the energy levels of the perovskite films for device structure design. Based on ultraviolet photoelectron spectroscopy (UPS), the Fermi level and valence band minimum for PEA15-SCN perovskite film (Fig. 2a) are calculated to be −4.52 eV and −5.08 eV, respectively. Combining the optical bandgaps of 1.39 eV for PEA15-SCN determined by Tauc plots (Supplementary Fig. 7b), the conduction band maximum of perovskite film is calculated to be −3.69 eV for PEA15-SCN (Fig. 2b). Considering the shallow conduction band position of tin perovskite, the typically used ETL, such as PCBM shows a large energy level offset due to its much deeper band position. Since the maximum attainable photovoltage is determined by the quasi-Fermi level splitting, $V_{{\mathrm{OC}}} = \frac{1}{q}\left( {E_{{\mathrm{Fn}}} - E_{{\mathrm{Fp}}}} \right)$, the use of shallower energy level ETL could increase the *V*_OC_ of the device^27^. Therefore, we used ICBA as ETL, since it has a LUMO level of −3.74 eV shallower than the commonly used PCBM of −3.91 eV (ref. ^28^).

### Device performance

**Fig. 3:**
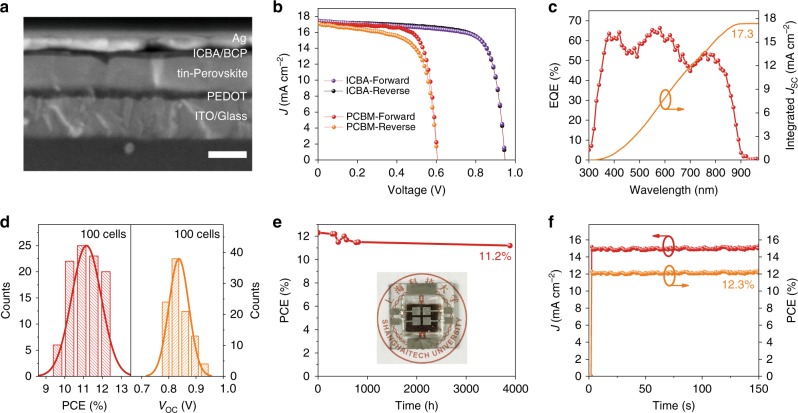
Photovoltaic performances of tin PSCs. **a** Cross-section SEM image of PEA15-SCN device. The scale bar is 200 nm. **b** *J–V* curves of the certified PEA15-SCN device with ICBA and champion device of PEA15-SCN film with PCBM. **c** EQE curve and integrated *J*_SC_ of the certified PEA15-SCN device. **d** Histograms for PCE and *V*_OC_ of PEA15-SCN device. **e** The stability of encapsulated PEA15-SCN device stored in N_2_ atmosphere. **f** Stabilized power output for the PEA15-SCN device (at 0.81 V) under simulated AM 1.5 G solar illumination at 100 mW cm^−2^.

We fabricated the device based on PEA15-SCN films above using ICBA and PCBM as ETL, respectively. The device based on ICBA achieved a much-enhanced voltage up to 0.94 V (Fig. 3a). The efficiency certified in an independent lab is up to 12.4% (*V*_OC_ *=* 0.94 V, *J*_SC_ = 17.4 mA cm^−2^, FF = 75%; Table 1, Supplementary Fig. 9). This is ~30% higher than the best value reported up to now^20–22^. In contrast, the device based on PCBM shows much worse efficiency of 7.7% (Fig. 3b, Supplementary Fig. 10), and the voltage is only 0.60 V. The device based on ICBA shows a low hysteresis as the current density–voltage (*J–V*) curves under reverse and forward scan overlapped well (Fig. 3b). The integrated photocurrent density (17.3 mA cm^−2^) obtaining from the external quantum efficiency (EQE) spectra of PEA15-SCN device (Fig. 3c) agrees closely with current density (*J*_SC_) in the *J–V* curves. Moreover, the device performance shows good reproducibility (Fig. 3d, Supplementary Fig. 11).

**Table 1:** Summary of the best performance devices with ICBA.

Device	PCE (%)	*V*_OC_ (V)	*J*_SC_ (mA cm^−2^)	FF (%)
PEDOT /PEA15/ICBA	10.1	0.78	17.8	72
PEDOT/PEA15-SCN/PCBM	7.7	0.60	17.1	74
PEDOT/PEA15-SCN/ICBA	12.4	0.94	17.4	75

We then fabricated devices based on PEA15 and ICBA for comparison, which shows *V*_OC_ of 0.78 V, much smaller than that the film with SCN (Table 1). Despite the current density of the film is comparable to that based on PEA15-SCN, the overall efficiency of the device is only 10.1%.

The PEA15-SCN device shows good shelf stability: the encapsulated device maintained 90% of the initial performance for as long as 3800 hours (Fig. 3e). Steady-state power conversion efficiency (PCE) measurement was carried out to evaluate the operation stability of the device. The device showed stable efficiency at continuous operation at maximum power point for 150 s (Fig. 3f). However, under longer time illumination, the efficiency decay slows, and the device loses 50% of its initial performance after continuous operation for over 300 min under amplitude modulation (AM) 1.5 illumination (Supplementary Fig. 13). The efficiency decay might be ascribed to the ion migration or accumulation of carriers at the interface.

### Device characterization

**Fig. 4:**
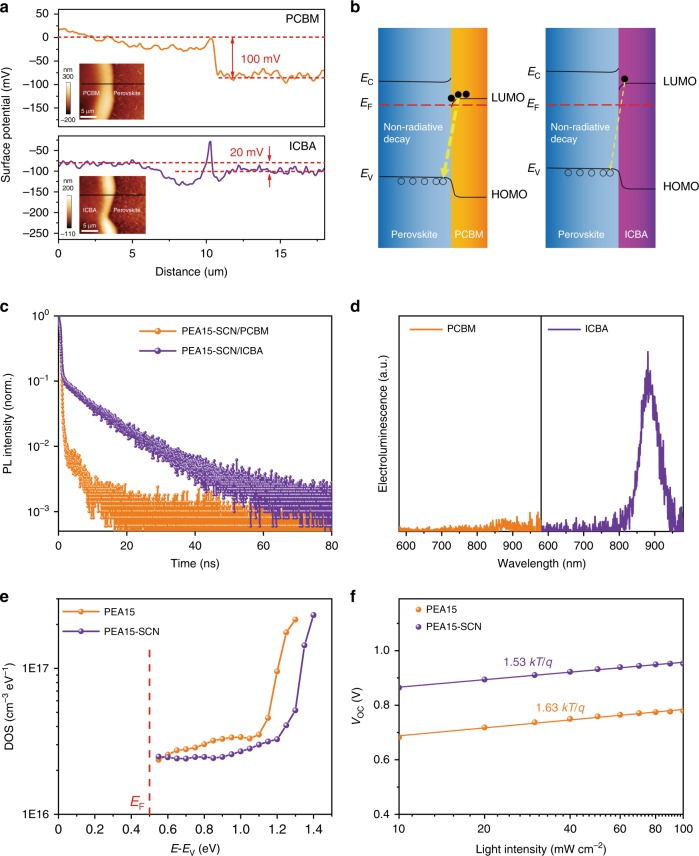
Recombination and defect density characterization. **a** Surface potential distribution of PEA15-SCN/PCBM and PEA15-SCN/ICBA from SKPM measurement. The insert images are AFM topography images for the corresponding samples. **b** The schematic diagrams of interface recombination for the two samples. **c** Time-resolved photoluminescence kinetics at 840 nm for the ITO/PEDOT/perovskite/ETL films after encapsulation. **d** Electroluminescence spectra of perovskite films under bias voltage of 2 V. **e** The density of states in the bandgap calculated from TPV and TPC. **f** *V*_OC_ versus illumination intensity for the devices.

To clarify the mechanism for the much-enhanced voltage and performance of the device based on ICBA, we studied the interface recombination between perovskite and ETL. Firstly, scanning Kelvin probe microscopy (SKPM) was employed to analyze interfacial energy level alignment of perovskite/ETL interface (Fig. 4a, Supplementary Figs. 14 and 15)^29^. The surface potential of ICBA is 20 mV higher than perovskite, indicating that the Fermi level of ICBA is 20 meV higher than that of perovskite. Similarly, we deduced that the Fermi level of PCBM is ~100 meV higher than perovskite (Fig. 4a). Hence, it can be calculated that the Fermi level of PCBM is 80 meV higher than that of ICBA. Considering that the LUMO level of PCBM is deeper than ICBA (Fig. 4b), the higher Fermi level of PCBM indicates that more states are occupied by electrons, i.e., it has higher electron density^27^. This can be attributed to remote doping from iodide, which can act as donor to inject electrons into ETL (ref. ^30^). In contrast, the shallow LUMO level of ICBA prohibits electron injection from iodide, giving rise to less electron density. The existence of iodide in ETL can be ascribed to ion migration in perovskite film^30^.

The high electron density of PCBM could aggravate interface carrier recombination with p-type tin perovskite film^31^. We hence performed time-resolved photoluminescence (TRPL) decay measurement to test the interface carriers recombination. The TRPL of different perovskite films shows a bi-exponential decay (Fig. 4c), including a fast component (*τ*_1_), and a slow component (*τ*_2_)^32^ (Table 2). The fast component can be ascribed to the rapid carriers recombination across the interfaces of perovskite and transporting layer^33^. The perovskite film contacting PCBM shows a smaller *τ*_1_, indicating faster carriers recombination at the interface. We used electroluminescence (EL) measurement under bias to estimate the interface recombination further^34,35^. As shown in Fig. 4d, PEA15-SCN device with ICBA shows an obvious EL peak, while extremely weak EL peak is observed for device with PCBM. The much higher EL intensity shows that the interface recombination is significantly suppressed, agreeing well with TRPL characterization.

**Table 2:** Decay time of PEA15-SCN film with ICBA and PCBM.

	*τ*_1_ (ns)	*τ*_2_ (ns)
ICBA	1.1	12.1
PCBM	0.25	2.2

We then used a model to quantitatively calculate the voltage loss due to interface carrier recombination. The *V*_OC_ loss $(\Delta V_{\rm{OC}}^{\rm{nrad}})$ of non-radiative decay at perovskite/ETL interface can be estimated by external radiative efficiency (ERE) from EL measurement based on the following equation^36^:

$$\Delta V_{{\mathrm{OC}}}^{{\mathrm{nrad}}} = \frac{{ - k_{\mathrm{B}}T}}{q}\ln {\mathrm{ERE}}$$

Where ERE is the electroluminescent EQE of the device. Based on the ERE of PEA15 and PEA15-SCN (Supplementary Fig. 16), the $\Delta V_{{\mathrm{OC}}}^{{\mathrm{nrad}}}$ are determined to be 417 mV and 226 mV for the device based on PCBM and ICBA, respectively. Hence, we conclude that interface recombination is another important factor that responsible for the low voltage of the device based on PCBM.

To investigate the function of the addition of NH_4_SCN in the film to device performances, we performed transient photovoltage (TPV) and transient photocurrent (TPC) measurements to test the density of defect states in the bandgap directly^37^ (Fig. 4e). The film with NH_4_SCN shows less density of defect states inside the bandgap, especially in the region close to the conduction band. We tested *V*_OC_ versus illumination intensity curves (Fig. 4f) and found that slope for the device based on PEA15-SCN is smaller than that based on PEA15, indicating the decrease of recombination of the film. Furthermore, the low Urbach energy of PEA15-SCN film in absorption spectra indicates the decrease of defect density as well. The reduced defect density of the film can be ascribed to the increase of domain size that reduced the grain boundary of the film, as discussed above. The reduced density of defects of SCN-treated film brings increased voltage and efficiency of the device.

## Discussion

In this work, we develop a device structure of tin PSCs using ICBA as ETL. The higher LUMO energy level of ICBA improves the maximum attainable voltage of the device and reduces the remote doping caused interface recombination. Furthermore, the use of NH_4_SCN as additive and PEDOT as hole transporting layer for perovskite growth reduces the defect density of the film. As a result, we are able to realize an unprecedentedly high voltage of 0.94 V for tin PSCs. Finally, we achieve a certified PCE up to 12.4%, which is almost 30% higher than the highest efficiency reported. With the open-circuit voltage approaching its theoretic limitation, this work indicates a huge potential for efficiency improvement of tin PSCs.

## Methods

### Device fabrication

SnI_2_, formamidinium iodide (FAI), phenethylammonium iodide (PEAI), and NiO_X_ were prepared according to literatures^12,13^. N,N-dimethylformamide (DMF), dimethyl sulfoxide (DMSO), chlorobenzene, isopropyl alcohol, SnF_2_, and NH_4_SCN were purchased from Sigma-Aldrich. Cleaned indium tin oxide (ITO) glass was treated in an ultraviolet ozone (UVO) machine for 20 min before fabrication. The PEDOT (Heraeus-Clevios P VP AI 4083) solution was spin coated onto ITO substrate at 6000 rpm for 60 s and then annealed at 140 °C for 20 min. A 0.8 M perovskite precursor (PEAI:FAI:SnI_2_:SnF_2_ = 0.15:0.85:1:0.075) and with or without NH_4_SCN (5 mol%) were added in mixed solvent (DMF:DMSO = 4:1 V/V) and stirred at 70 °C for 1 h. The precursor was spin coated at 1000 rpm and 5000 rpm for 10 s and 30 s, respectively. And 600 μL toluene (Sinopharm Chemical Reagent Co. Ltd., redistilled) was dropped during the second process at eighth second. The substrate was then annealed at 70 °C for 10 min. The films of PEA15 and PEA0 were made by the same procedure. A total of 18 mg mL^−1^ ICBA (1-Material) or PCBM (nano-C) in chlorobenzene was spin coated at 1000 rpm for 30 s, and then annealed at 70 °C for 10 min. Saturated bathocuproine in isopropyl alcohol was spin coated at 6000 rpm for 30 s, followed by annealing at 70 °C for 10 min. All precursors were filtered with 0.22 μm polytetrafluoroethylene (PTFE) filters before spin coated. Finally, 100 nm Ag layer was deposited under vacuum of <10^−7^ torr using an Angstrom Engineering deposition system.

### Film characterizations

XRD was recorded on a Bruker D8 Advance using Cu Kα source (*λ* = 1.54 Å). PL spectra were carried out by the exciting wavelength at 480 nm of spectrofluorometer (Fluorolog; HORIBA FL-3) with a standard 450 W Xenon CW lamp. TRPL spectroscopy at 840 nm was measured using Fluorolog HORIBA FL-3 with a pulsed source at 504 nm DeltaDiode DD-510L. The UV–vis spectra of the perovskite films deposited on PEDOT were recorded by UV–vis spectrophotometer (Agilent cary5000). SEM images were recorded on JSM-7800. UPS was performed for films on PEDOT using a Thermo Fisher ESCALAB 250XI, and the samples were transferred from glovebox to vacuum chamber with a portable transfer capsule. Curve fitting was performed using the Thermo Avantage software. The curves were corrected based on the C1s peak at 284.8 eV. GIWAXS studies were performed at the BL16B1 beamline of Shanghai Synchrotron Radiation Facility (SSRF), Shanghai, China, using beam energy of 10 keV (*λ* = 1.24 Å) and a Mar 225 detector. The grazing-incidence angles for all films were 0.2° and 1.0°, respectively. GIXGUI Matlab toolbox was utilized for necessary corrections of GIWAXS raw patterns and collecting the azimuth angle^38^.

### Device characterization

*J*–*V* curves were measured using a Keithley 2400 source unit under simulated AM 1.5 G solar illumination at 100 mW cm^−2^ (1 sun). The light intensity was calibrated by means of a KG-5 Si diode with a solar simulator (Enli Tech, Taiwan). The devices are measured in reverse scan (1.0–0 V, step 0.01 V) and forward scan (0–1.0 V, step 0.01 V) with a delay time of 30 ms. The *J*–*V* curves for all devices were measured by masking the active area using a metal mask with an area of 0.04 cm^2^. The steady-state PCE was performed at 0.81 V. Devices were stored and tested in the same nitrogen-filled glovebox (Vigor).

The EQE spectra were measured by a commercial system (Solar cell scan 100, Beijing Zolix Instruments Co., Ltd). The cells were subjected to monochromatic illumination (150 W Xe lamp passing through a monochromator and appropriate filters). The light intensity was calibrated by a standard photodetector (QE-B3/S1337-1010BQ, Zolix). The light beam was chopped at 180 Hz, and the response of the cell was acquired by a Stanford Research SR830 lock-in amplifier.

TPV and TPC measurements were carried out using a setup comprising a 532 nm wavelength laser to provide steady-state bias light, a 640 nm wavelength laser, and an oscilloscope. A power-adjustable 532 nm wavelength laser was used to get a steady *V*_OC_ of the device. A 640 nm diode laser was used to modulate the *V*_OC_ on top of a constant light bias. The pulse duration was set to 1 μs and the repetition rate to 50 Hz by the function generator of the oscillator. The digital oscilloscope recorded the data induced by the light perturbation, using 1 MΩ input impedance for the TPV measurement and 50 Ω impedance for TPC measurement. We determined the charge generated (Δ*Q*) in the devices by integrating the TPC curve by the 640 nm laser pulse without 532 nm light bias present. The calculated *C* = Δ*Q*/Δ*V*_OC_ result is the capacitance. The total charge carrier was obtained by integrating the *C* versus *V*_OC_. The carrier concentration (*n*) for each open-circuit voltage was calculated by dividing each charge carriers with the device volume. The density of states (DOS) in the mid-gap can be obtained by differentiating the carrier density with respect to the *V*_OC_ following a previously reported procedure^37^.

The current density–luminance–radiance (*J*–*V*–*R*) characteristics were measured by a Keithley 2400 source meter, and a fiber integrating sphere (FOIS-1) couple with a QE Pro 650 spectrometer (Ocean Optics). The devices were tested on top of the integrating sphere, and only forward light emission could be collected. All device test processes were carried out in the N_2_-filled glovebox.

### First-principles calculations

First-principles calculations were performed within the framework of DFT using plane-wave pseudopotential methods, as implemented in the Vienna Ab-initio Simulation Package^39^. The generalized gradient approximation formulated by Perdew, Burke, and Ernzerhof was used as the exchange–correlation functional. The electron–core interactions were described by the projector augmented-wave^40^ method for the pseudo potentials. The cutoff energy for the plane-wave basis set used to expand the Kohn–Sham orbitals was 400 eV. The Gamma-centered *k*-point mesh with a grid spacing of 2π × 0.03 Å^−1^ was used for electronic Brillouin zone integration. For adsorption energy calculation, the vacuum thickness was set to be 20 Å. The equilibrium structural parameters (including both lattice parameters and internal coordinates) of each involved bulk material were obtained via total energy minimization by using the conjugate gradient algorithm, with the force convergence threshold of 0.01 eV Å^−1^; for slab structures, only the internal coordinates are relaxed. The optB86b-vdW (ref. ^41^) functional is adopted throughout the whole calculation.

### SKPM measurement

The amplitude-modulation SKPM was operated combined with a Cypher S atomic force microscopy (AFM; Asylum Research, Oxford Instruments) and a HF2LI Lock-in amplifier (Zurich Instruments) in N_2_-filled glovebox. The resonance frequency *ω*_0_ and spring constant of AFM conducting tips are ~127 kHz and 5.0 Nm^−1^, respectively.

### Tin PSC certification at SIMIT (Shanghai, China)

The device certification tests were performed at an independent lab (SIMIT, Chinese Academy of Sciences). And SIMIT is accredited by China National Accreditation Service for Conformity Assessment (CNAS) to ISO/IEC 17025 and by the International Laboratory Accreditation Cooperation (ILAC) Mutual Recognition Arrangement.

A silicon reference solar cell (PVM1211, NREL_ISO tracking#:1974) was used to set the irradiance at 100 mW cm^−2^ at standard testing conditions in accordance with IEC 60904-3 ed.2 AM 1.5 G. *J*–*V* characteristics of tin PSCs were measured under simulated sunlight by steady-state class AAA solar simulator according to IEC 60904-9 ed.2. The spectral mismatch was calculated and mismatch correction was performed according to IEC 60904-7 ed.3. The *J*–*V* curves were measured in forward and reverse scans with a scanning speed of 90 mV s^−1^.

### Encapsulation method

Devices were encapsulated by quartz glass and UV-glue for *J*–*V*, EQE, TRPL, TPV, and TPC measurements. Films were double sealed by plastic bags before XRD, SEM, PL, UV–vis, and GIWAXS measurements.

### Reporting summary

Further information on experimental design is available in the Nature Research Reporting Summary linked to this paper.

## Supplementary information

Supplementary Information

Solar Cells Reporting Summary

## Data Availability

The data that support the findings of this study are available from the corresponding author upon reasonable request.
