# Supplementary material for: Ultra-high open-circuit voltage of tin perovskite solar cells via an electron transporting layer design
Source: Nat Commun. 2020 Mar 6;11:1245. doi: 10.1038/s41467-020-15078-2 (PMC7060347; doi:10.1038/s41467-020-15078-2)
Supplement: Supplementary file 1 — Supplementary Information [file 41467_2020_15078_MOESM1_ESM.pdf]

Supplementary Information

Ultra-high open-circuit voltage of tin perovskite solar cells induced via an electron transporting layer design

Jiang et al.

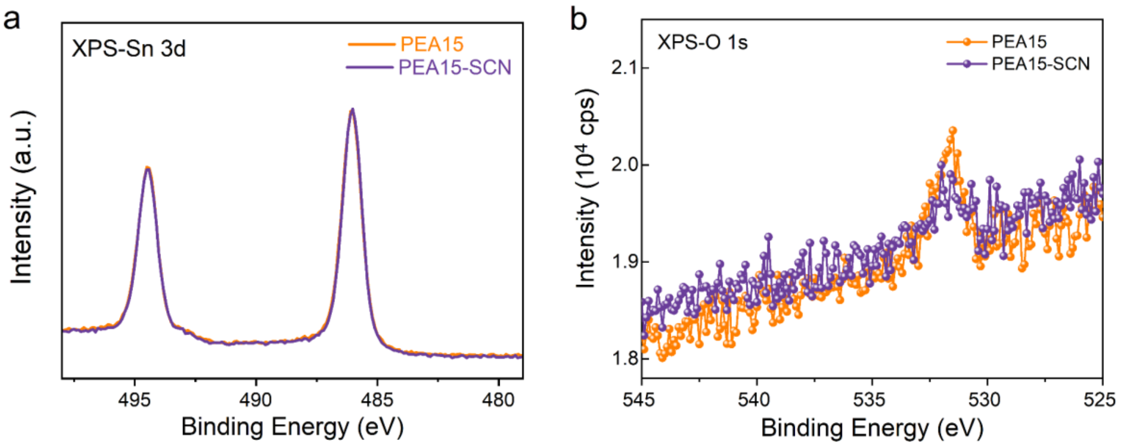

Supplementary Figure 1 XPS Sn 3d (a) and XPS O 1s (b) spectra of perovskite films deposited on ITO/PEDOT substrates. No obvious peak difference is observed for the two samples.

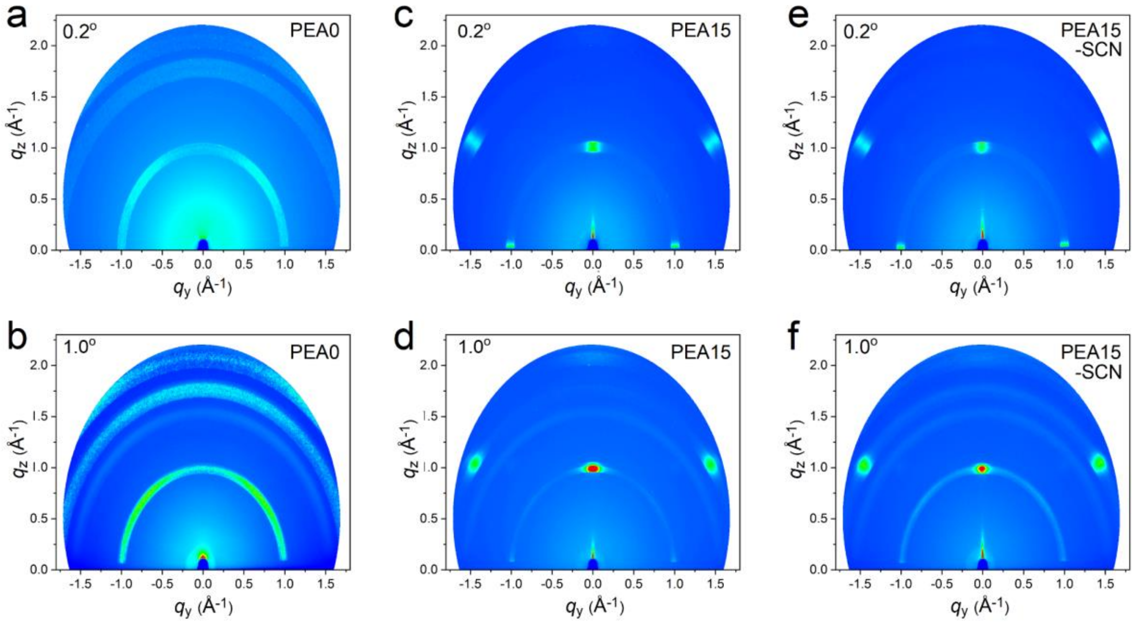

Supplementary Figure 2 The entirety GIWAXS images PEA0 (a-b), PEA15 (c-d), and PEAI15-SCN (e-f) perovskite films at incident angles of 0.2° and 1.0°.

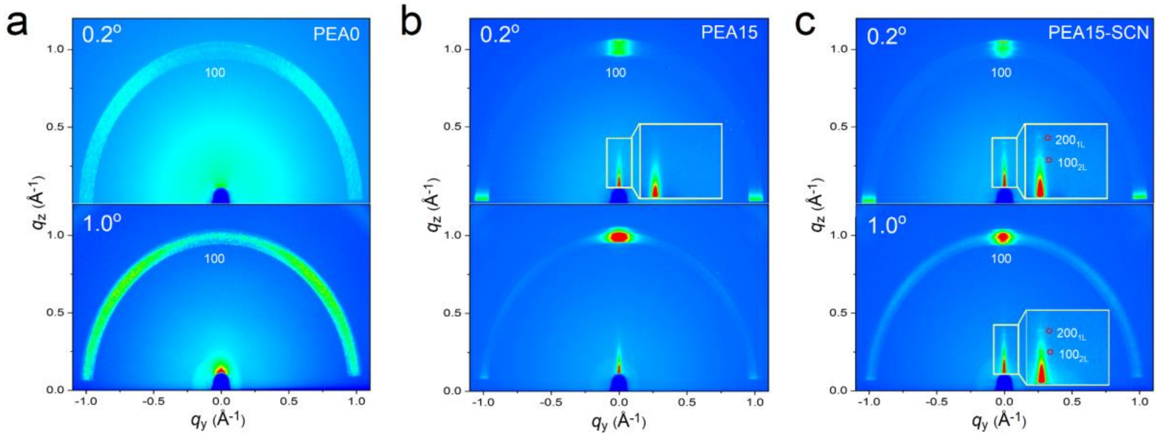

Supplementary Figure 3 GIWAXS images of PEA0 (a), PEA15 (b), and PEA15-SCN (c) perovskite films at incident angles of 0.2° and 1.0°, respectively.

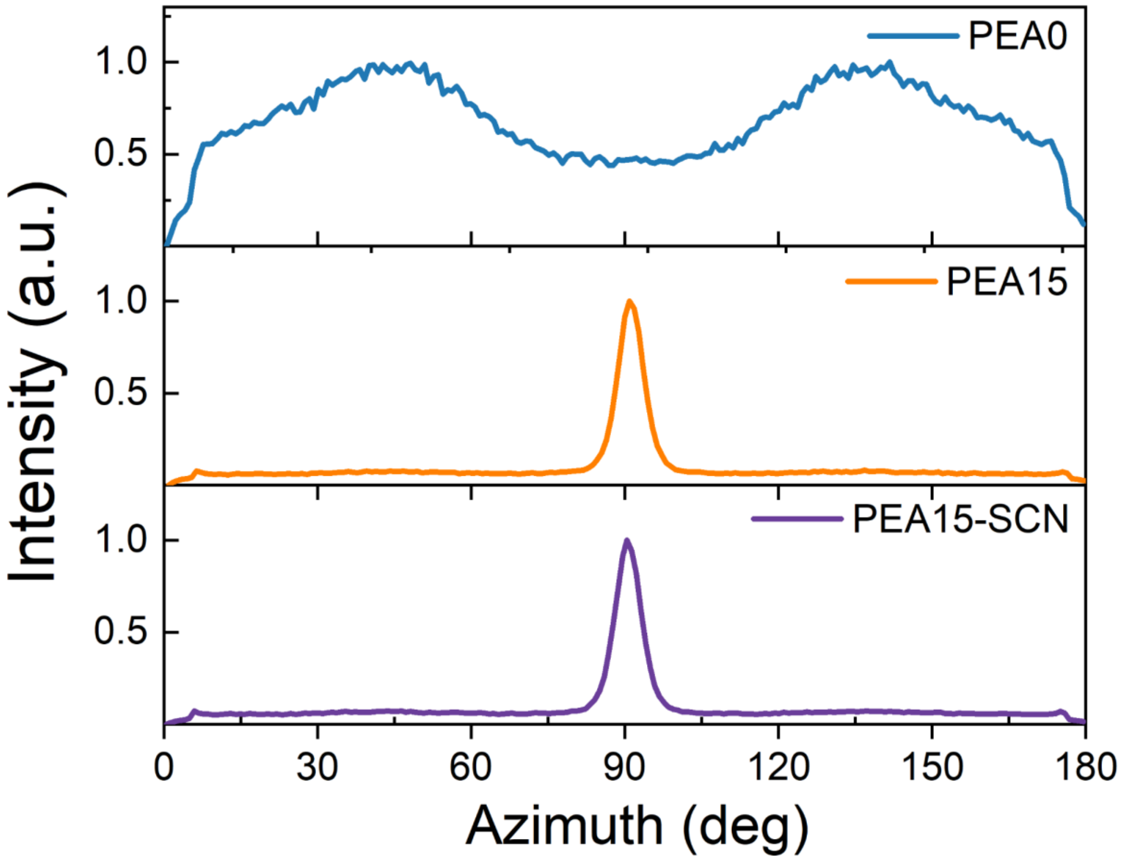

Supplementary Figure 4 Integrated intensity plots azimuthally along the ring at q_r approximate to 1.0 Å^{-1} based on 1.0° GIWAXS patterns, assigned to the (100) plane of perovskite films.

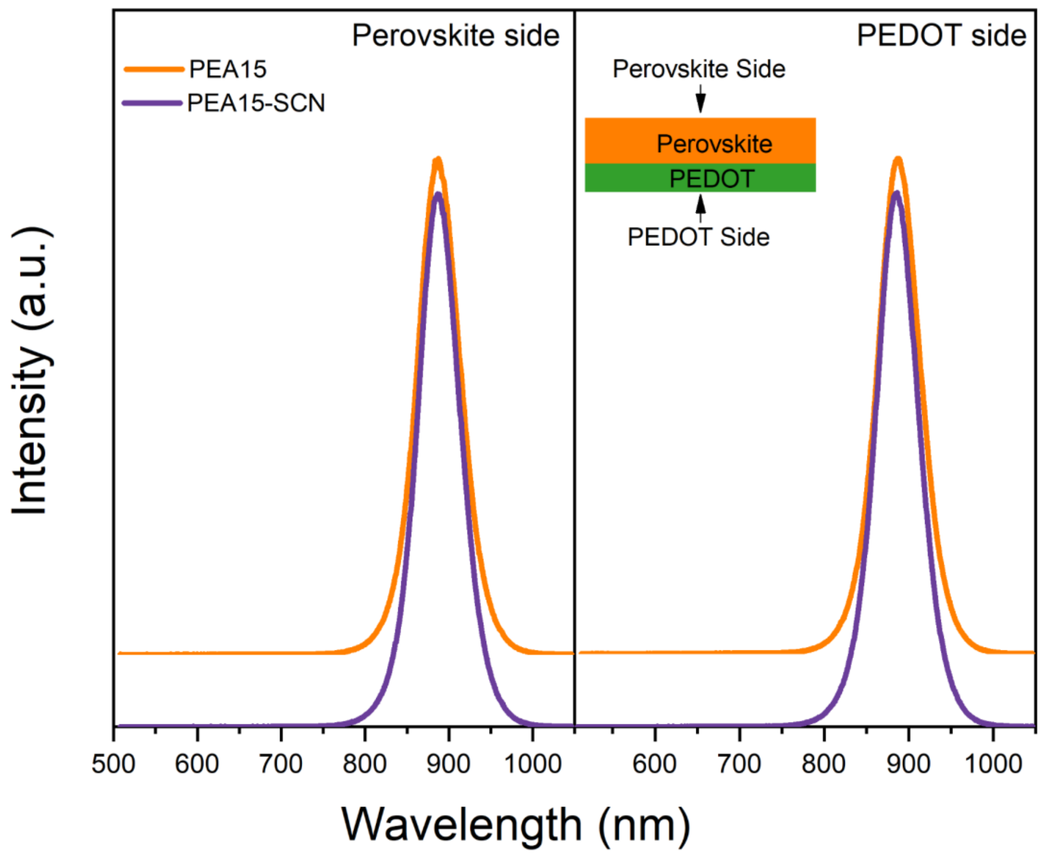

Supplementary Figure 5 PL spectra of different perovskite films when excited from both perovskite and PEDOT sides. The inset picture is the schematic of perovskite and PEDOT sides.

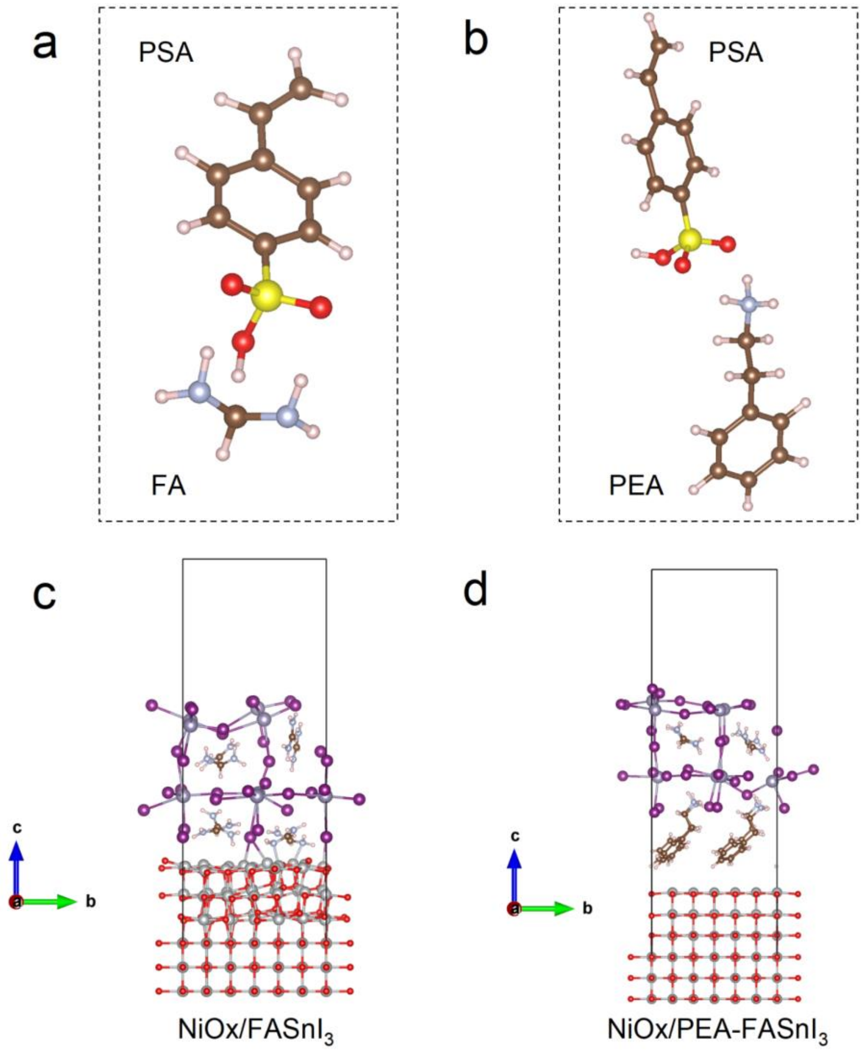

Supplementary Figure 6 Structure models for binding energy calculation of (a) PSA (2-phenylethenesulfonic acid) and FA, (b) PSA and PEA, (c) NiO_X (001) and $FASnI_3$ (001), (d) NiO_X (001) and PEA-$FASnI_3$ (001).

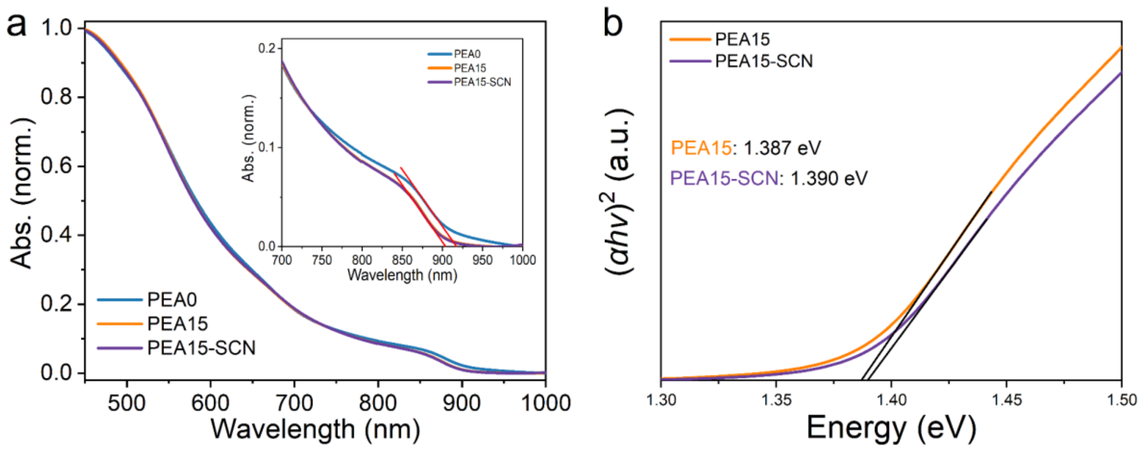

Supplementary Figure 7 UV-vis spectra (a) and Tauc plots (b) of perovskite films.

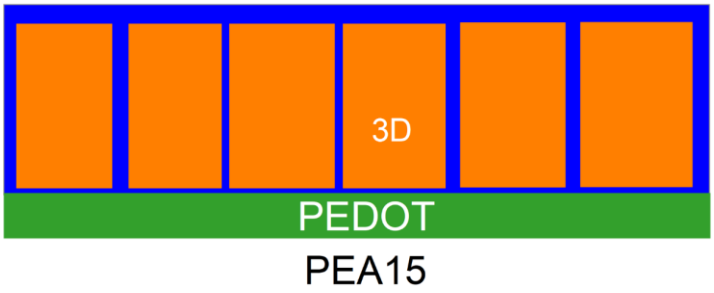

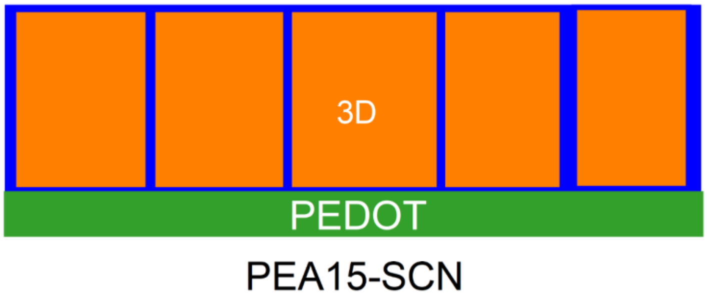

Supplementary Figure 8 Schematic illustration of PEA15 and PEA15-SCN perovskite film structures based on PEDOT substrates.

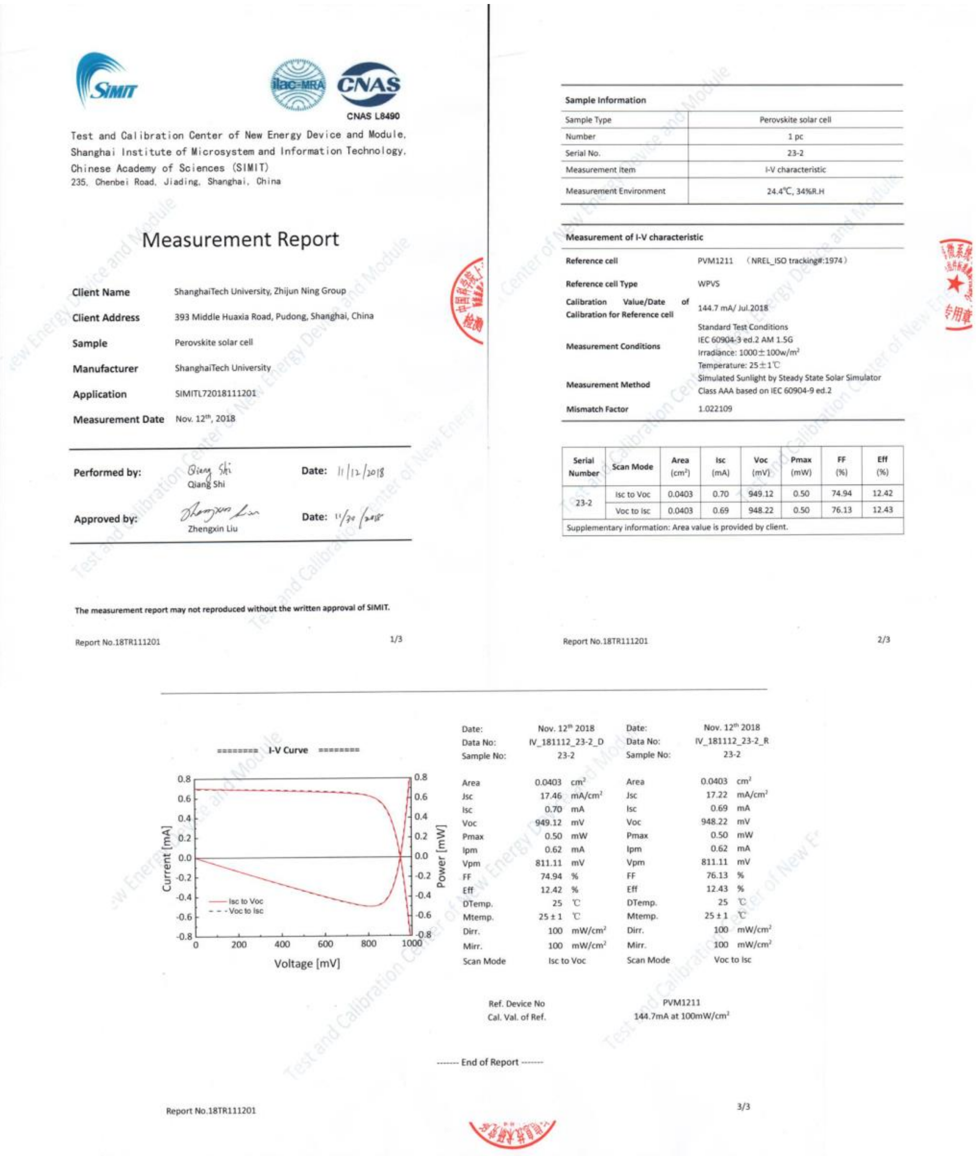

CNAS L8490

Test and Calibration Center of New Energy Device and Module,
Shanghai Institute of Microsystem and Information Technology,
Chinese Academy of Sciences (SIMIT)
235, Chenbei Road, Jiading, Shanghai, China

Measurement Report

**Client Name**	ShanghaiTech University, Zhijun Ning Group
**Client Address**	393 Middle Huaxia Road, Pudong, Shanghai, China
**Sample**	Perovskite solar cell
**Manufacturer**	ShanghaiTech University
**Application**	SIMITL72018111201
**Measurement Date**	Nov. 12th, 2018

Performed by: Qiang Shi **Date:** 11/12/2018

Approved by: Zhengxin Liu **Date:** 11/20/2018

The measurement report may not reproduced without the written approval of SIMIT.

Report No.18TR111201 1/3

Sample Information

Sample Type	Perovskite solar cell
Number	1 pc
Serial No.	23-2
Measurement Item	I-V characteristic
Measurement Environment	24.4℃, 34%R.H

Measurement of I-V characteristic

**Reference cell**	PVM1211 （NREL_ISO tracking#:1974）
**Reference cell Type**	WPVS
**Calibration Value/Date of Calibration for Reference cell**	144.7 mA/ Jul.2018
**Measurement Conditions**	Standard Test Conditions<br>IEC 60904-3 ed.2 AM 1.5G<br>Irradiance: 1000±100w/m²<br>Temperature: 25±1℃
**Measurement Method**	Simulated Sunlight by Steady State Solar Simulator Class AAA based on IEC 60904-9 ed.2
**Mismatch Factor**	1.022109

Serial Number	Scan Mode	Area (cm²)	Isc (mA)	Voc (mV)	Pmax (mW)	FF (%)	Eff (%)
23-2	Isc to Voc	0.0403	0.70	949.12	0.50	74.94	12.42
	Voc to Isc	0.0403	0.69	948.22	0.50	76.13	12.43
Supplementary information: Area value is provided by client.							

Report No.18TR111201 2/3

Date:	Nov. 12th 2018		Date:	Nov. 12th 2018	
Data No:	IV_181112_23-2_D		Data No:	IV_181112_23-2_R	
Sample No:	23-2		Sample No:	23-2	
Area	0.0403	cm²	Area	0.0403	cm²
Jsc	17.46	mA/cm²	Jsc	17.22	mA/cm²
Isc	0.70	mA	Isc	0.69	mA
Voc	949.12	mV	Voc	948.22	mV
Pmax	0.50	mW	Pmax	0.50	mW
Ipm	0.62	mA	Ipm	0.62	mA
Vpm	811.11	mV	Vpm	811.11	mV
FF	74.94	%	FF	76.13	%
Eff	12.42	%	Eff	12.43	%
DTemp.	25	℃	DTemp.	25	℃
Mtemp.	25 ± 1	℃	Mtemp.	25 ± 1	℃
Dirr.	100	mW/cm²	Dirr.	100	mW/cm²
Mirr.	100	mW/cm²	Mirr.	100	mW/cm²
Scan Mode	Isc to Voc		Scan Mode	Voc to Isc	

Ref. Device No PVM1211
Cal. Val. of Ref. 144.7mA at 100mW/cm²

------- End of Report -------

Report No.18TR111201 3/3

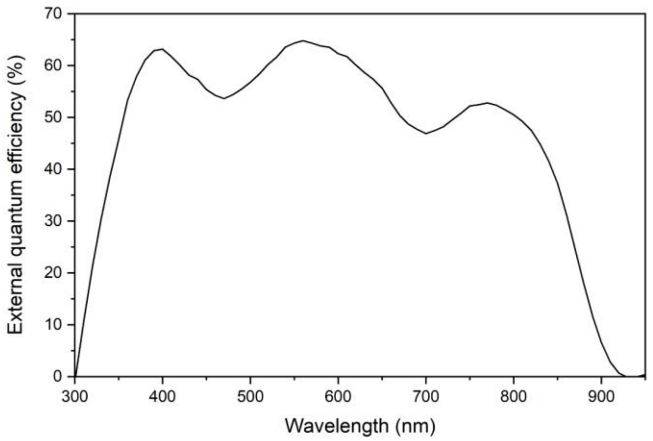

Supplementary Figure 9 Certification data including *J*-*V* curves and external quantum efficiency spectrum of the device based on PEA15-SCN by Certification Centre of the New Energy Device and Module at SIMIT.

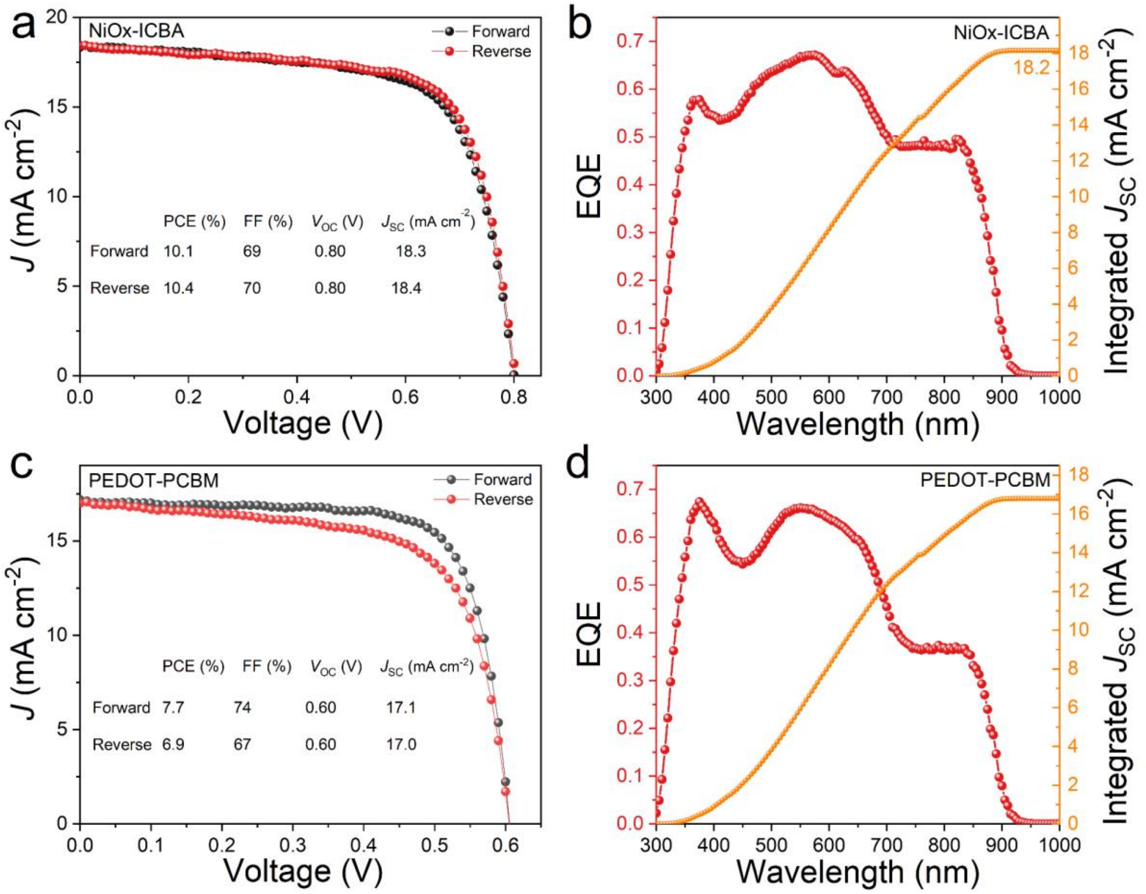

Supplementary Figure 10 *J*-*V* and EQE curves of PEA15-SCN with highest V_{OC}: NiO_X as HTL and ICBA as ETL (a-b); PEDOT as HTL and PCBM as ETL (c-d). Device of PEA15-SCN based on NiO_X substrate was used to study the impact of substrate. The device using ICBA as ETL generates a V_{OC} of 0.80 V and an efficiency around 10%, higher than that based on PCBM as well[1], confirming the role of ICBA in elevating V_{OC}. The relatively low efficiency of the device based on NiO_X compared to that based on PEDOT could be ascribed to its hierarchy structure hindered effective carriers injection into ICBA. The higher current density of the device based on NiO_X may derive from its high hole mobility and better carriers extraction[2].

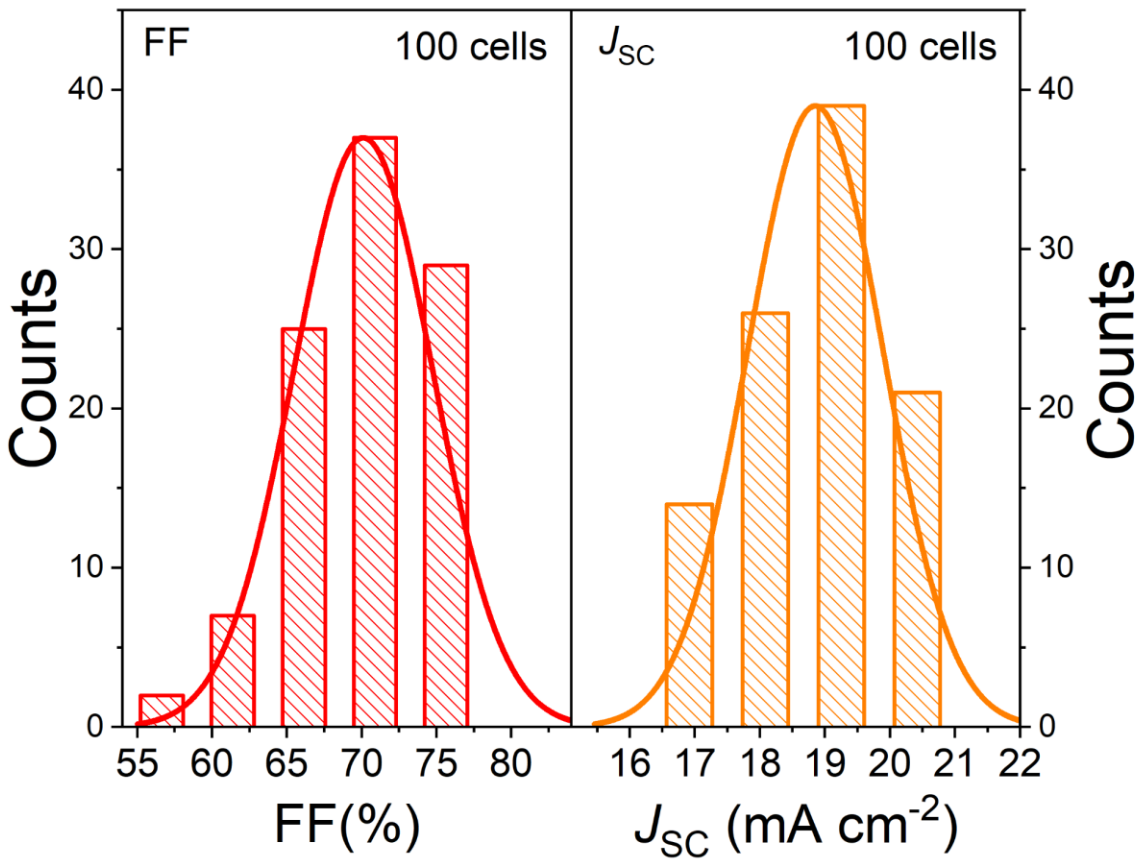

Supplementary Figure 11 Histograms for (a) FF and (b) J_{SC} of PEA15-SCN device: PEDOT as HTL and ICBA as ETL.

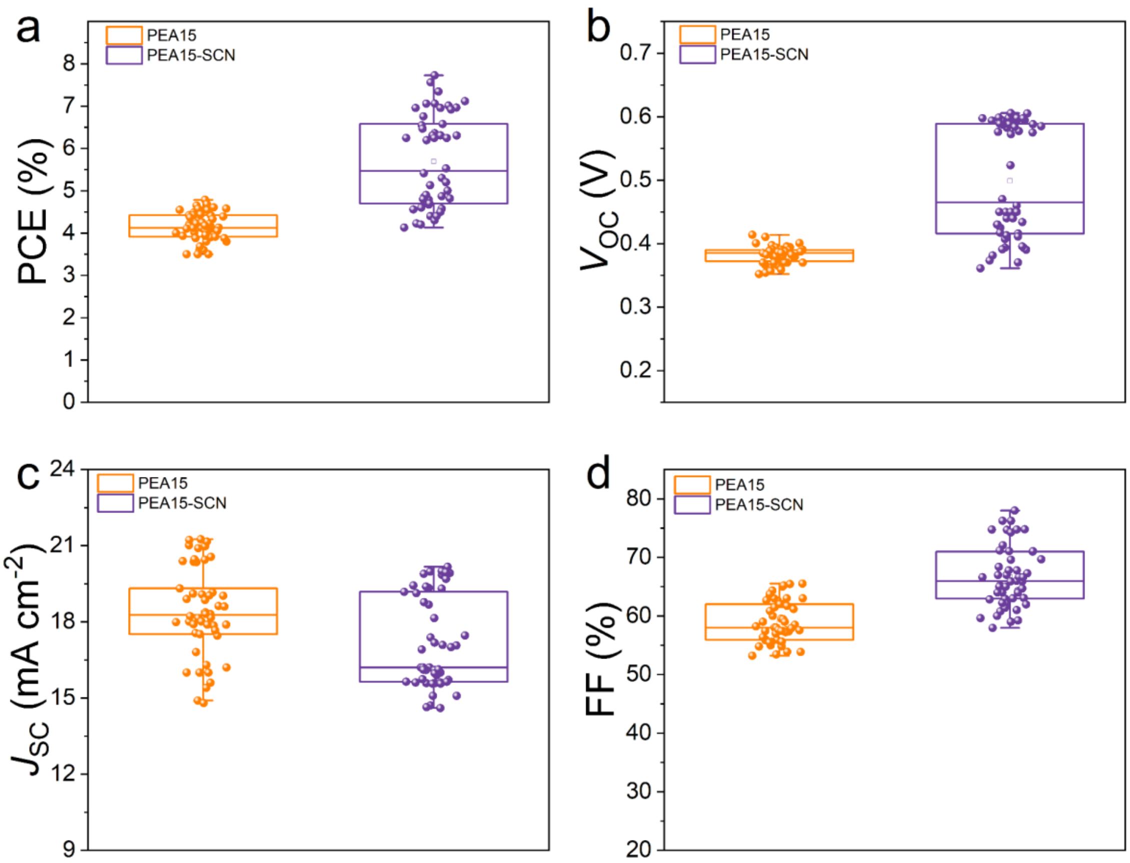

Supplementary Figure 12 Device parameters of perovskite using PEDOT as HTL and PCBM as ETL: (a) PCE, (b) V_{OC}, (c) J_{SC}, and (d) FF.

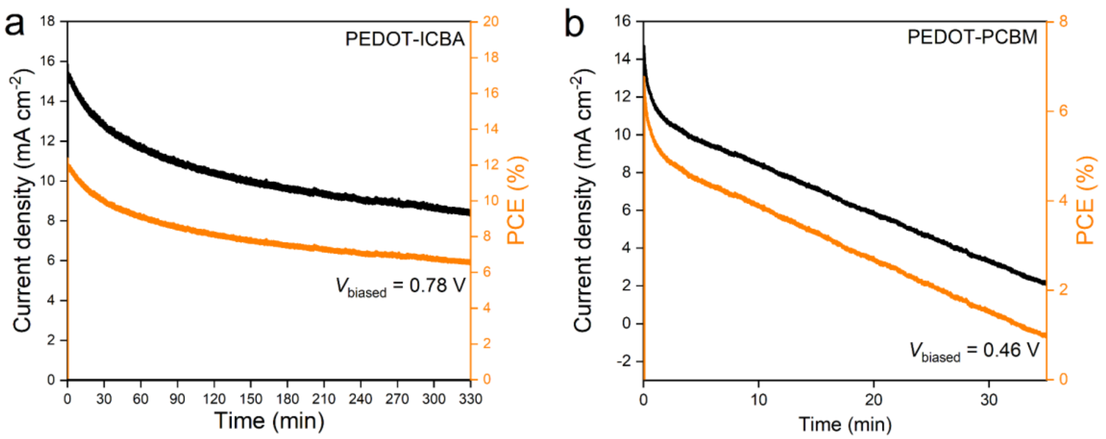

Supplementary Figure 13 Stabilized power output for the devices under simulated AM 1.5G solar illumination (100 mW cm^{-2}) using (a) ICBA and (b) PCBM as ETL, respectively. The poor stability of the device using PCBM as ETL can be ascribed to the density of iodine vacancy at the interface.

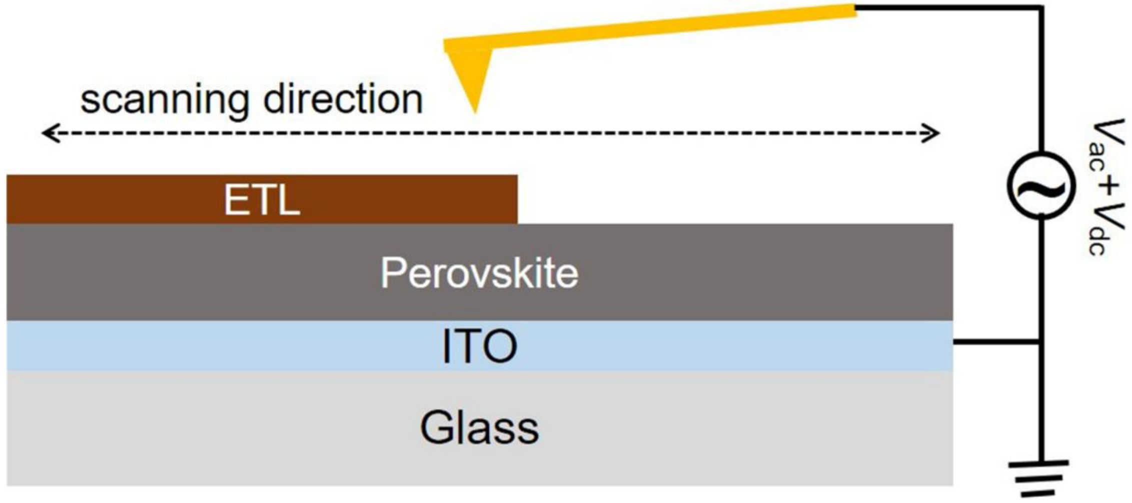

Supplementary Figure 14 Schematic of SKPM measurements.

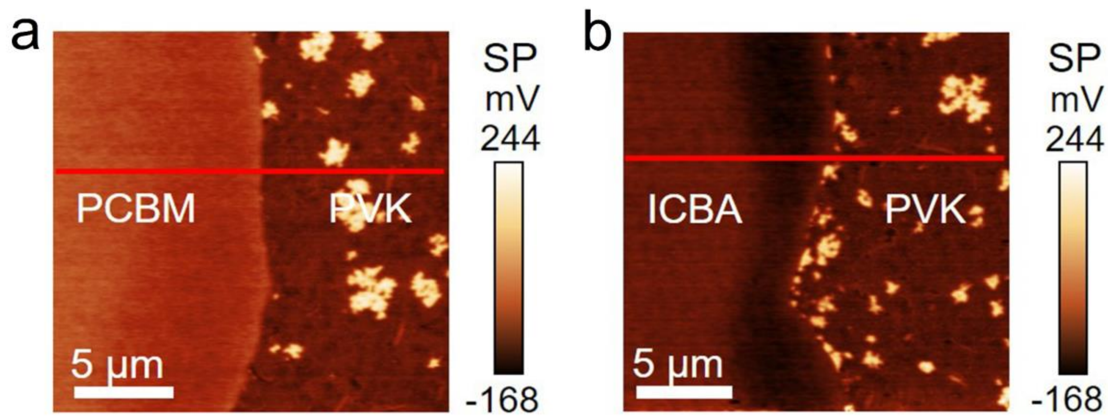

Supplementary Figure 15 AFM surface potential characterization of SKPM measurements for PEA15-SCN/PCBM (a) and PEA15-SCN/ICBA (b).

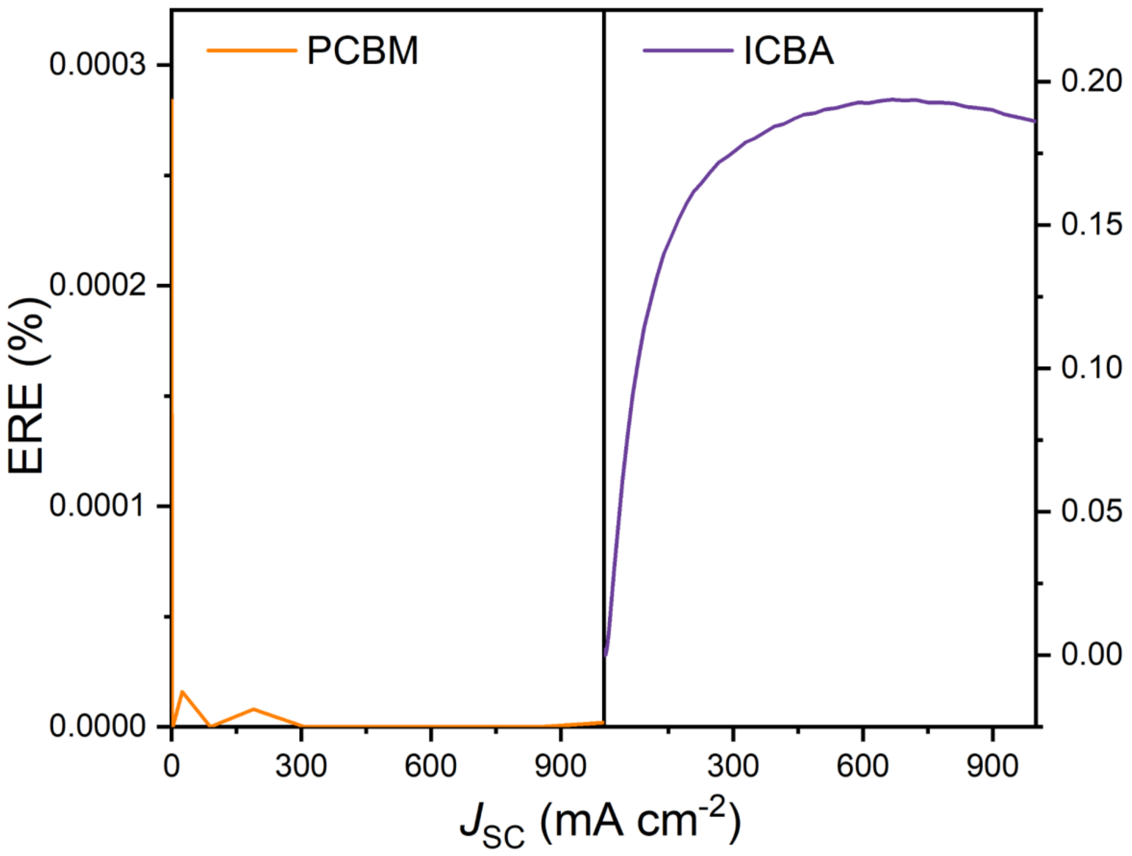

Supplementary Figure 16 External radiative efficiency (ERE) spectra of PEA15-SCN devices.

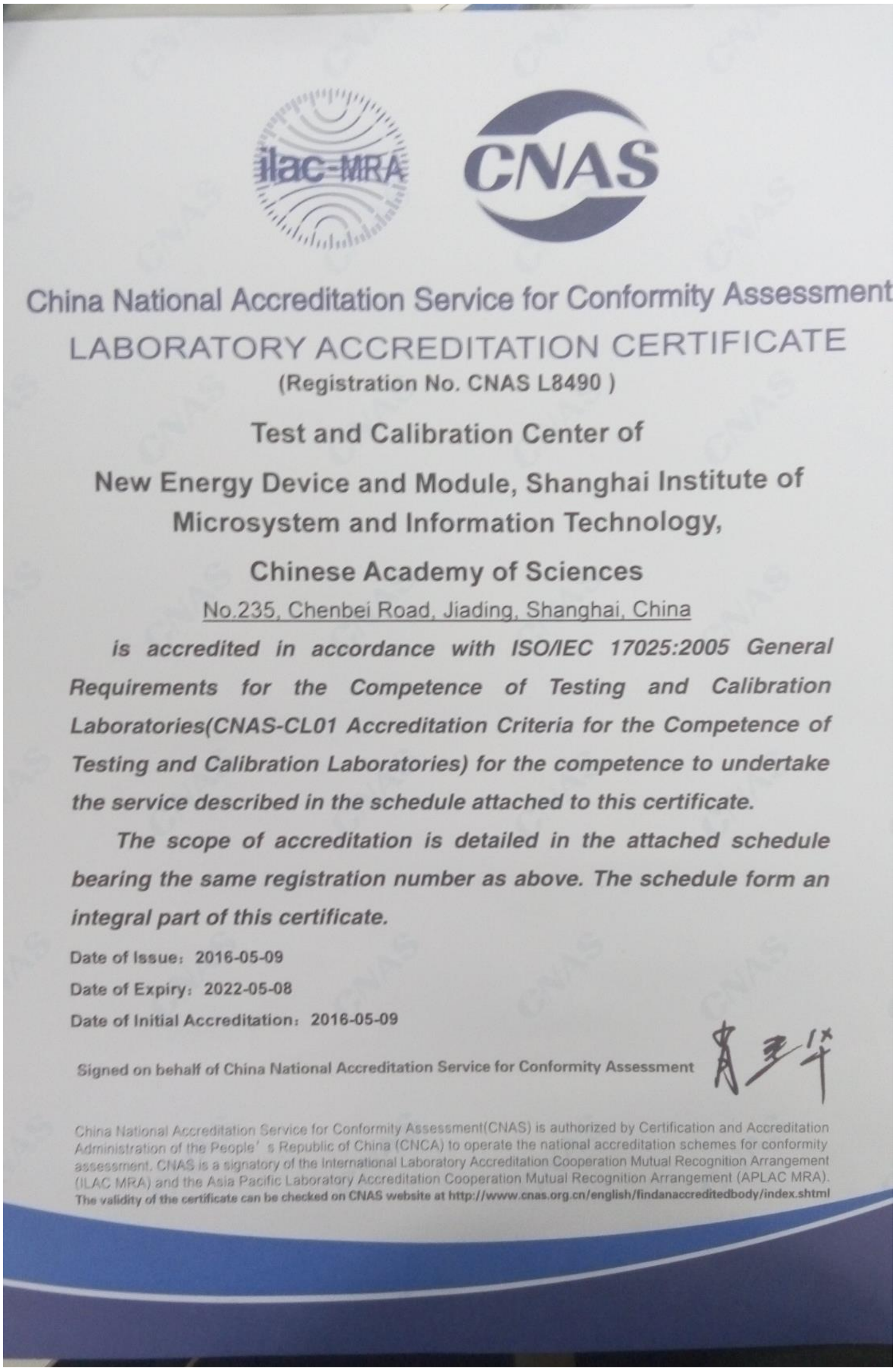

China National Accreditation Service for Conformity Assessment

LABORATORY ACCREDITATION CERTIFICATE

(Registration No. CNAS L8490)

Test and Calibration Center of

New Energy Device and Module, Shanghai Institute of Microsystem and Information Technology,

Chinese Academy of Sciences

No.235, Chenbei Road, Jiading, Shanghai, China

is accredited in accordance with ISO/IEC 17025:2005 General Requirements for the Competence of Testing and Calibration Laboratories(CNAS-CL01 Accreditation Criteria for the Competence of Testing and Calibration Laboratories) for the competence to undertake the service described in the schedule attached to this certificate.

The scope of accreditation is detailed in the attached schedule bearing the same registration number as above. The schedule form an integral part of this certificate.

Date of Issue：2016-05-09

Date of Expiry：2022-05-08

Date of Initial Accreditation：2016-05-09

Signed on behalf of China National Accreditation Service for Conformity Assessment

China National Accreditation Service for Conformity Assessment(CNAS) is authorized by Certification and Accreditation Administration of the People' s Republic of China (CNCA) to operate the national accreditation schemes for conformity assessment. CNAS is a signatory of the International Laboratory Accreditation Cooperation Mutual Recognition Arrangement (ILAC MRA) and the Asia Pacific Laboratory Accreditation Cooperation Mutual Recognition Arrangement (APLAC MRA). The validity of the certificate can be checked on CNAS website at http://www.cnas.org.cn/english/findanaccreditedbody/index.shtml

Supplementary Figure 17 The qualification certificate from CNAS of the SIMIT certification center.

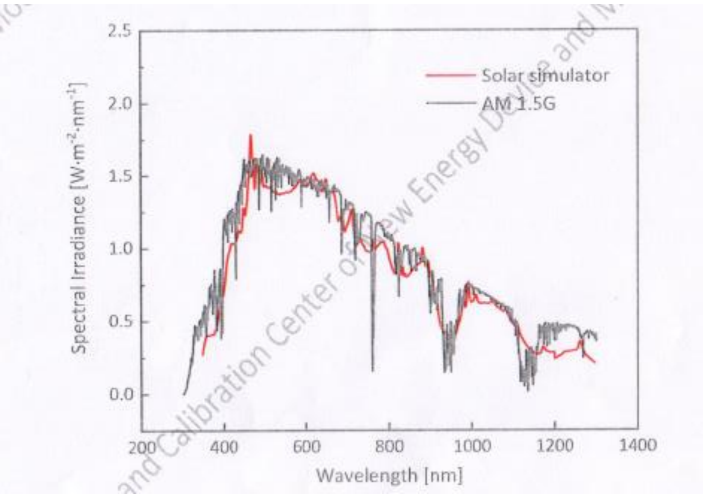

Supplementary Figure 18 The solar simulator spectral distribution produced by a dual-lamp (xenon and halogen) class AAA solar simulator used by SIMIT certification center.

Supplementary Table 1 DFT calculation of adsorption energy

Interaction items	Adsorption energy
PSA and PEA	-2.321 eV
PSA and FA	-2.427 eV
NiO_X (100) slab and PEA	-2.355 eV
NiO_X (100) slab and FA	-7.393 eV
NiO_X (100) and $FASnI_3$ (100)	-122.6 meV $Å^{-2}$
NiO_X (100) and PEA-$FASnI_3$ (100)	-53.8 meV $Å^{-2}$

Supplementary Note 1:

The calculation of different binding energy. Considering that the ratio of PEDOT and PSS in PEDOT:PSS is about 1:6, we used the monomer of PSS, PSA, to represent the surface structure of PEDOT:PSS.

The binding energy was calculated according to the following equation:

$$E_{\mathrm{b}} = E_{\mathrm{tot}} - E_{\mathrm{sub}} - E_{\mathrm{ads}} \qquad \text{(\textbf{Supplementary Equation 1})}$$

where E_{b} is the binding energy, E_{tot} is the energy of the whole structure, E_{sub} is the energy of the substrate, E_{ads} is the energy of the adsorption, and the binding energy per unit area for the interface of NiO_X (001) and $FASnI_3$ (001) or PEA-$FASnI_3$ (001) was calculated as follows:

$$E_{\mathrm{area}} = \frac{E_{\mathrm{b}}}{S} \qquad \text{(\textbf{Supplementary Equation 2})}$$

where E_{area} is the binding energy per unit area, E_{b} is the calculated binding energy, S is the area of the interface.

Supplementary Note 2:

The certification tests of device. The certification tests were performed by the Test and Calibration Centre of the New Energy Device and Module, SIMIT, Chinese Academy of Sciences, which is accredited by China National Accreditation Service for Conformity Assessment (CNAS) to ISO/IEC 17025 and by the International Laboratory Accreditation Cooperation (ILAC) Mutual Recognition Arrangement. CNAS was the accreditation body member of the International Accreditation Forum and ILAC, and is also a member of the Asia Pacific Laboratory Accreditation Cooperation and Pacific Accreditation Cooperation.

The *J-V* characteristics were measured using a steady-state class AAA dual-lamp (xenon and halogen) super solar simulator under standard test conditions according to IEC 60904-1:2006. The *J–V* curves were measured in forward and reverse scans with a scanning speed of 90 mV s^{-1}. A World PV Scale (WPVS) silicon reference solar cell calibrated by NREL was used to set the irradiance at 100 mW cm^{-2}. The spectral mismatch was calculated and mismatch correction was performed according to IEC 60904-7:2008. The spectral irradiance of the solar simulator was measured by a calibrated spectroradiometer (Opto Research Corporation), and the EQE of the device under test was measured with a three-grating monochromator spectral response measurement system.

Supplementary References

1. Wang, F. *et al.* 2D-Quasi-2D-3D Hierarchy Structure for Tin Perovskite Solar Cells with Enhanced Efficiency and Stability. *Joule* **2**, 2732-2743 (2018).
2. You, J.B. *et al.* Improved air stability of perovskite solar cells via solution-processed metal oxide transport layers. *Nat. Nanotechnol.* **11**, 75-78 (2016).
