# Supplementary material for: Ultra-high open-circuit voltage of tin perovskite solar cells via an electron transporting layer design
Source: Nat Commun. 2020 Mar 6;11:1245. doi: 10.1038/s41467-020-15078-2 (PMC7060347; doi:10.1038/s41467-020-15078-2)
Supplement: Supplementary file 2 — Solar Cells Reporting Summary [file 41467_2020_15078_MOESM2_ESM.pdf]

Corresponding author(s): Zhijun Ning

Solar Cells Reporting Summary

Nature Research wishes to improve the reproducibility of the work that we publish. This form is intended for publication with all accepted papers reporting the characterization of photovoltaic devices and provides structure for consistency and transparency in reporting. Some list items might not apply to an individual manuscript, but all fields must be completed for clarity.

For further information on Nature Research policies, including our data availability policy, see Authors & Referees.

ü Experimental design

Please check: are the following details reported in the manuscript?

1. Dimensions

Item	Yes	No	Details
Area of the tested solar cells	☒	☐	Methods in the manuscript
Method used to determine the device area	☒	☐	Methods in the manuscript

2. Current-voltage characterization

Item	Yes	No	Details
Current density-voltage (J-V) plots in both forward and backward direction	☒	☐	Fig. 3b
Voltage scan conditions<br>*For instance: scan direction, speed, dwell times*	☒	☐	Methods in the manuscript
Test environment<br>*For instance: characterization temperature, in air or in glove box*	☒	☐	Methods in the manuscript
Protocol for preconditioning of the device before its characterization	☒	☐	Methods in the manuscript
Stability of the J-V characteristic<br>*Verified with time evolution of the maximum power point or with the photocurrent at maximum power point; see ref. 7 for details.*	☒	☐	Figure 3f, Supplementary Figure 13

3. Hysteresis or any other unusual behaviour

Item	Yes	No	Details
Description of the unusual behaviour observed during the characterization	☐	☒	Not relevant
Related experimental data	☐	☒	Not relevant

4. Efficiency

Item	Yes	No	Details
External quantum efficiency (EQE) or incident photons to current efficiency (IPCE)	☒	☐	Figure 3c
A comparison between the integrated response under the standard reference spectrum and the response measure under the simulator	☒	☐	Methods in the manuscript
For tandem solar cells, the bias illumination and bias voltage used for each subcell	☐	☒	single solar cell

5. Calibration

Item	Yes	No	Details
Light source and reference cell or sensor used for the characterization	☒	☐	Methods in the manuscript
Confirmation that the reference cell was calibrated and certified	☒	☐	Methods in the manuscript

Calculation of spectral mismatch between the reference cell and the devices under test

- [x] Yes — It has been calibrated
- [ ] No

6. Mask/aperture

Size of the mask/aperture used during testing

- [x] Yes — Methods in the manuscript
- [ ] No

Variation of the measured short-circuit current density with the mask/aperture area

- [x] Yes — Methods in the manuscript
- [ ] No

7. Performance certification

Identity of the independent certification laboratory that confirmed the photovoltaic performance

- [x] Yes — SIMIT
- [ ] No

A copy of any certificate(s)
Provide in Supplementary Information

- [x] Yes — Supplementary Figure 9
- [ ] No

8. Statistics

Number of solar cells tested

- [x] Yes — Figure 3d
- [ ] No

Statistical analysis of the device performance

- [x] Yes — Figure 3d
- [ ] No

9. Long-term stability analysis

Type of analysis, bias conditions and environmental conditions
For instance: illumination type, temperature, atmosphere humidity, encapsulation method, preconditioning temperature

- [x] Yes — Figure 3e
- [ ] No
